# Oncolytic properties of non-vaccinia poxviruses

**DOI:** 10.18632/oncotarget.26288

**Published:** 2018-11-13

**Authors:** Marine Ricordel, Johann Foloppe, Christelle Pichon, Annie Findeli, Caroline Tosch, Pascale Cordier, Sandrine Cochin, Eric Quémeneur, Christelle Camus-Bouclainville, Stéphane Bertagnoli, Philippe Erbs

**Affiliations:** ^1^ Transgene SA, Illkirch-Graffenstaden 67405, France; ^2^ IHAP, INRA, Université de Toulouse, Toulouse 31058, France; ^3^ Current address: Polyplus-transfection SA, Illkirch-Graffenstaden 67400, France

**Keywords:** non-vaccinia poxviruses, oncolytic properties, RCNtk-/gfp::fcu1

## Abstract

Vaccinia virus, a member of the *Poxviridae* family, has been extensively used as an oncolytic agent and has entered late stage clinical development. In this study, we evaluated the potential oncolytic properties of other members of the *Poxviridae* family. Numerous tumor cell lines were infected with ten non-vaccinia poxviruses to identify which virus displayed the most potential as an oncolytic agent. Cell viability indicated that tumor cell lines were differentially susceptible to each virus. Raccoonpox virus was the most potent of the tested poxviruses and was highly effective in controlling cell growth in all tumor cell lines. To investigate further the oncolytic capacity of the Raccoonpox virus, we have generated a thymidine kinase (TK)-deleted recombinant Raccoonpox virus expressing the suicide gene *FCU1*. This TK-deleted Raccoonpox virus was notably attenuated in normal primary cells but replicated efficiently in numerous tumor cell lines. In human colon cancer xenograft model, a single intratumoral inoculation of the recombinant Raccoonpox virus, in combination with 5-fluorocytosine administration, produced relevant tumor growth control. The results demonstrated significant antitumoral activity of this new modified Raccoonpox virus armed with *FCU1* and this virus could be considered to be included into the growing armamentarium of oncolytic virotherapy for cancer.

## INTRODUCTION

Oncolytic virotherapy for cancer treatment utilizes naturally occurring or engineered viruses for selective infection and cancer cell death without any adverse effect on normal cells. Vaccinia virus (VACV) is one of the best studied representative of the *Orthopoxvirus* genus. Its potency to preferentially infect and kill cancer cells has been proven for many years and advanced clinical trials are ongoing to evaluate this virus in human [1]. Numerous preclinical and clinical trials have been performed in a variety of cancer indications using several strains of oncolytic VACV including Wyeth, Western Reserve, Lister, and Copenhagen strains [2–6]. The Wyeth based vector Pexa-Vec (JX-594) has shown efficacy in hepatocarcinoma (HCC) [1] and a Phase 3 study is ongoing in primary HCC (National Clinical Trial NCT02562755). Like Pexa-Vec, most of the oncolytic VACVs reported to date encode mutations that inactivated the TK, a critical enzyme in the salvage pathway for nucleotide biosynthesis. Cellular TK expression is generally decreased in normal cells, but increased in rapidly proliferating tumor cells [7]. The TK-deleted VACV can selectively infect tumor tissues, whereas in most normal cells, deletion of the *TK* gene greatly reduces the virus replication [8].

A few other viruses of the *Poxviridae* family have been studied for their potential oncolytic properties; this assessment includes Myxoma, Yaba-like disease, Raccoonpox, ORF and Cowpox viruses [9–13]. Despite these studies, our knowledge is limited regarding the potential oncolytic properties of other members of the *Poxviridae* family. Here, we explored the oncolytic capacities of ten non-vaccinia poxviruses regarding their effect on tumor cell proliferation and tumor growth control.

*The Poxviridae* family is subdivided into two sub-families: the *Entomopoxvirinae* infecting strictly insects and the *Chordopoxvirinae*, infecting a large range of vertebrates. The latter is sub-divided into at least nine genera including *Parapoxvirus, Orthopoxvirus, Leporipoxvirus, Suipoxvirus, Avipoxvirus, Yatapoxvirus* and unassigned poxvirus [14].

Parapoxviruses (PPVs) include ORF virus (ORFV), Bovine papular stomatitis virus (BPSV), and Pseudocowpox virus (PCPV) [15]. Features that distinguish PPVs from other poxvirus genera are the ovoid virion shape, the crisscross pattern on the particle surface, and the relatively small size and high GC content of the genome [16]. PPVs cause non-systemic, eruptive skin disease in domestic and wild mammals. ORFV, the prototype species of PPVs, is responsible for contagious ecthyma, an acute disease of sheep and goats. The disease is characterized by proliferative lesions in the skin of the lips and in the oral mucosa. Lesions progress through a typical pattern of erythema, papula, pustule, scab and usually resolve in 1 to 2 months. High mortality rates occur when lesions in lips and udders prevent infected animals from suckling and grazing, resulting in rapid emaciation [17]. ORFV has been well described as vaccine vector for veterinary issues [18] but also as oncolytic vector for viral therapy against cancer [9]. Preclinical studies have confirmed previous *in vitro* results and presented ORFV as an alternative for vaccinia virus platform [9].

BPSV infects cattle of all ages but clinical signs are usually seen in calves. The disease has a worldwide distribution and is characterized by papules, often mildly erosive, on the muzzle, oral mucosa, and udder and occasionally in the esophagus and forestomach [19]. Like ORFV in sheep and goats, reinfection of cattle with BPSV is commonly observed, suggesting that virus infection does not confer significant immunity.

PCPV infects cattle worldwide with zoonotic potential. The infection is most frequent in milking herds, affecting the teats and udder of cows and the muzzles and mouths of nursing calves. The lesions of pseudocowpox are characterized by “ring” or “horseshoe”-shaped scabs, the latter being characteristic of the disease. Infection is transmitted by cross-suckling of calves, improperly disinfected teat clusters of milking machines, and probably by the mechanical transfer of virus by flies [19]. PCPV can infect the unprotected hands of people working with affected cattle, causing “milker’s nodules” [20].

Yaba-like disease virus (YLDV) belongs to the genus *Yatapoxvirus* and causes vesicular skin lesions in primates [21, 22], although the natural reservoir of this virus is uncertain. This virus was first recognized in monkey caretakers in primate centers in the United States [23]. YLDV infection in caretakers produced a brief fever and the development of a few necrotic maculopapular nodules, followed by complete resolution of the infection. A TK-deleted YLDV expressing GFP was constructed and was investigated as an replicating poxvirusfor cancer gene therapy [11]. This recombinant YLDV demonstrated, *in vitro*, a 3-log expansion over 96 hr in human tumor cell lines and an *in vivo* efficiency of tumor gene delivery in mice with a human ovarian tumor model.

Myxoma virus (MYXV) is the type species of the *Leporipoxvirus* genus. The virus naturally infects the South American tapeti, causing a cutaneous fibroma at the inoculation site. However, in the European rabbit, which is exotic to the Americas, MYXV causes myxomatosis with high mortality rates but it is totally harmless in humans [24]. Recent preclinical studies demonstrate that MYXV is an attractive oncolytic virus candidate for treating various human cancers [25].

The Squirrel Fibroma virus (SQFV), another member of the *Leporipoxvirus* genus, induces cutaneous fibromas and proliferative epidermal lesions in eastern gray squirrels in North America [26, 27]. Generalized disease can occur in suckling squirrels with proliferative lesions over the body and in the lungs, liver, lymph nodes and kidney [26, 27]. Infection was readily transmitted from infected young squirrels to juveniles by mosquitoes, but adult squirrels were difficult to infect and virus titres in the resulting fibromas were not sufficient for mosquito transmission [27]. However, fibromas and generalized disease have been reported in naturally infected adult squirrels [27, 28].

Raccoonpox virus (RCNV) is a member of the *Orthopoxvirus* genus and is closely related to the Vaccinia and Cowpox viruses [29]. RCNV was first isolated in 1961 from a naturally occurring infection from the respiratory tract of raccoons [30]. This virus has no known pathology in any mammalian species including raccoons. *In vitro* data demonstrated replication of RCNV in most human tumor cells from the NCI-60 cancer cell panel and RCNV treatment significantly delayed the progression of solid tumors in both xenograft and syngeneic tumor models [10].

Fowlpox virus (FPV), the prototypical member of the *Avipoxvirus* genus, infects chickens and turkeys and causes moderate pathology in poultry. Recombinant FPV vaccines expressing foreign antigens have been used to immunize animals against avian and mammalian diseases [31]. Because FPV undergoes abortive replication in mammalian cells, its use as a safe vehicle for expression of foreign antigens and host immunomodulators has been evaluated in numerous clinical trials of vaccines against cancer, malaria, tuberculosis and AIDS [32].

Swinepox virus (SWPV) belongs to the *Suipoxvirus* genus. It is the etiologic agent of a skin disease of pigs, characterized by generalized pustular lesions and associated with high rates of illness. Swinepox disease has a worldwide distribution and is mechanically transmitted by pig lice or through direct animal contact [33]. The potential of using recombinant SWPV as a porcine vaccine candidate against infectious diseases of domestic pigs was evaluated [34].

Cotia virus (CTV) is an unclassified poxvirus isolated from arbovirus sentinel laboratory mice in South America. A natural host has not been identified, but the virus is presumably insect transmitted. It seems more closely related to *Capripoxvirus*, *Suipoxvirus*, *Yatapoxvirus*, *Leporipoxvirus*, and *Cervidpoxvirus* [35].

The purpose of the present study was to determine *in vitro* the spectrum of efficacy of these ten poxviruses in a variety of different cancer cells. RCNV, the most effective virus, was subsequently armed with the *FCU1* gene [36]. *FCU1* encodes a bifunctional fusion protein with combined cytosine deaminase and uracil phosphoribosyltransferase activity which converts the non-toxic 5-fluorocytosine (5-FC) into the clinically approved chemotherapeutic 5-fluorouracil (5-FU) and further into 5-fluorouracil-monophosphate (5-FUMP), which leads to inhibition of DNA and protein synthesis [36]. The *FCU1* gene was inserted into the *TK* locus of the RCNV genome under the control of the strong p11k7.5 promoter. *In vitro*, we assessed the ability of the recombinant RCNV to replicate and kill various human tumor cell lines. We also evaluated the behavior of the recombinant RCNV on human primary cells.

We further examined the anti-tumor effect of this TK-deleted virus *in vivo*. We demonstrated here that RCNV is more effective than other non-vaccinia poxviruses and possesses the features of a novel oncolytic platform, amenable to be armed with therapeutic transgenes.

## RESULTS

### Oncolytic activity of the ten poxviruses and progeny virion production of poxviruses

**Figure 1:**
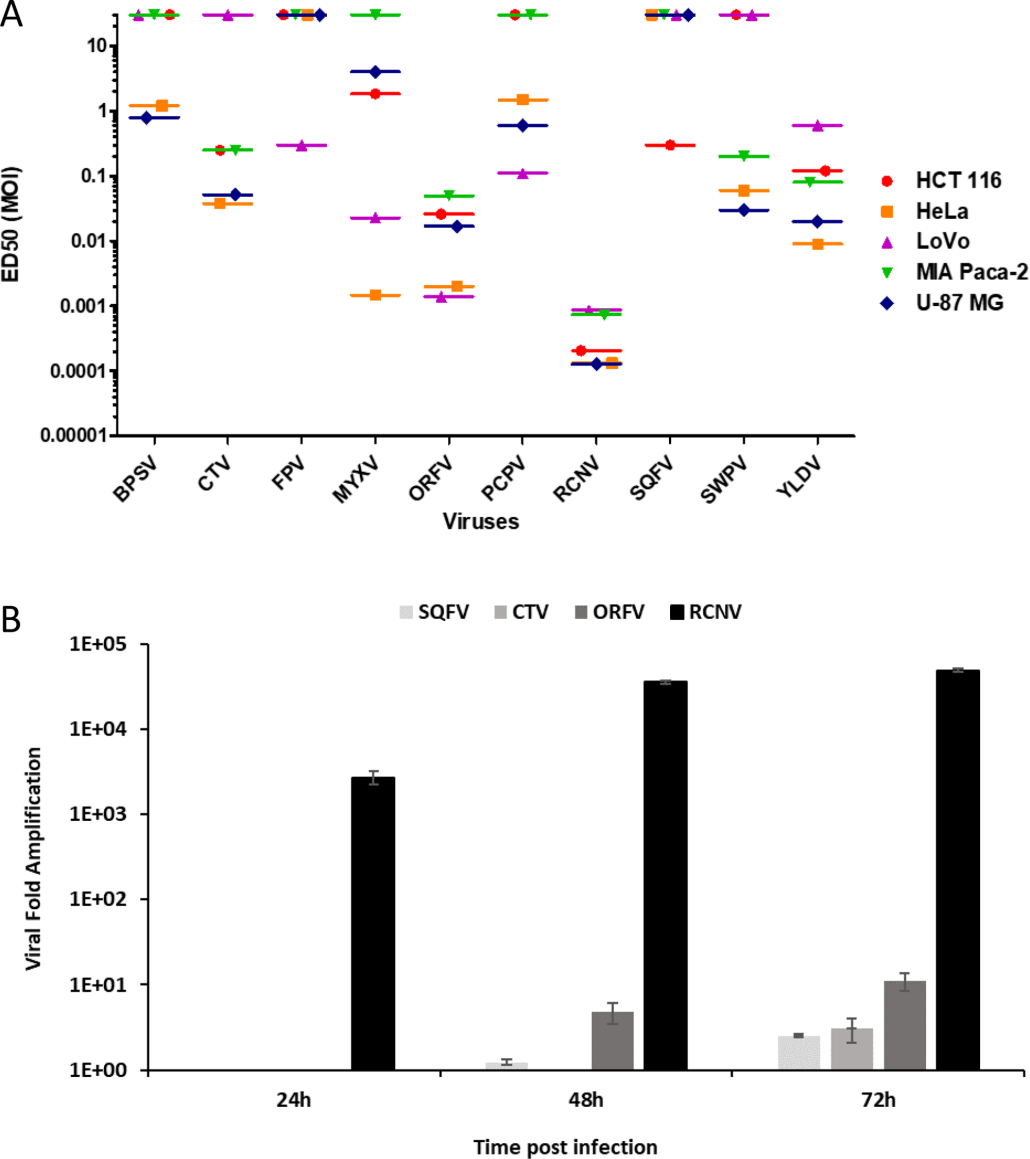
Comparison of different poxviruses in human tumor cell lines and progeny virion production (**A**) ED50 (MOI in pfu/cell) on a panel of tumor cell lines upon poxviruses infection at multiple MOI. Five human cancer cell lines were infected with the different viruses at several MOI from 1 to 10^–5^. Viability was evaluated 5 days later by trypan blue exclusion. Each experiment was done in triplicate. ED50 was determined by using the MOI which allow 50% of cell death. ED50 values greater than 10 indicate that tumor cells were insensitive to the virus at the tested MOIs. (**B**) Replication of RCNV, ORFV, SQFV and CTV on human glioblastoma cell line. U-87 MG cells were infected at MOI 0.01 in 6 well plates. At each time point, cells were harvested, sonicated and titrated on adequate cells. Results are expressed as viral fold increased (corresponding to output/input ratio).

To evaluate the relative ability of the different poxviruses to control the growth of tumor cells, the ED50 of the 10 poxviruses was assessed on a panel of cancer cell lines of different origins (Figure 1A).

Even at the highest used MOI (MOI 1), FPV and SQFV did not show any effect on the proliferation, except a weak cytotoxic effect in LoVo and HCT 116 cells for FPV and SQFV, respectively. The other poxviruses demonstrated varying efficacy in different cell lines. For example, SWPV and CTV were effective at MOI 10^–1^ in U-87 MG and HeLa cells but weakly effective in LoVo, HCT 116 and MIA PaCa-2 cell lines. ORFV exhibited the best growth control in LoVo and HeLa cells but was moderately effective in MIA PaCa-2, HCT 116 and U-87 MG cells. MYXV was most effective in HeLa cells at an MOI of 10^–3^, and in a lesser extent in LoVo cells (ED50 of 2 × 10^–2^). Among all the viruses tested, RCNV exhibited the best control of cellular proliferation across all cancer cell lines tested. Low concentrations of RCNV (≤MOI of 10^–3^) were effective at reducing cell viability of human tumor cell lines of various tissue origins. Nevertheless, there was some variation in the sensitivity of cells to RCNV. For example, RCNV was very effective in U-87 MG, HeLa and HCT 116 cells (ED50 of 10^–4^) and less effective in MIA PaCa-2 and LoVo cells with an ED50 of 10^–3^.

In order to extend our results and to assess the ability of the experimental poxviruses to complete their life cycle once they had entered cells and produce infectious progeny, we performed a replication assay in the human glioblastoma U-87 MG cell line with four of the poxviruses (RCNV, ORFV, SQFV and CTV) having a different oncolytic activity in U-87 MG cell line (Figure 1A).

As shown in Figure 1B, RCNV was more productive in U-87 MG cell line than ORFV, SQFV and CTV. RCNV replication reached 30,000-fold increase 48 h post infection. Lower amplification was shown using ORFV with only 10-fold increase 72 h post infection. The replication of CTV was extremely low with only 3-fold increase three days post infection and SQFV produced less than 2-fold increase even after 72 h of infection. RCNV could produce a large quantity of viral particles in 72 h compared to three other representatives of the *Poxviridae* family (ORFV, SQFV and CTV). Knowing that there is a correlation between the ability of a virus to display so called oncolytic activity and its ability to multiply in tumor cells, this replication assay confirms that RCNV is clearly the most potent oncolytic virus among these different poxviruses tested.

### Construction of a TK-deleted and armed Raccoonpox virus

**Figure 2:**
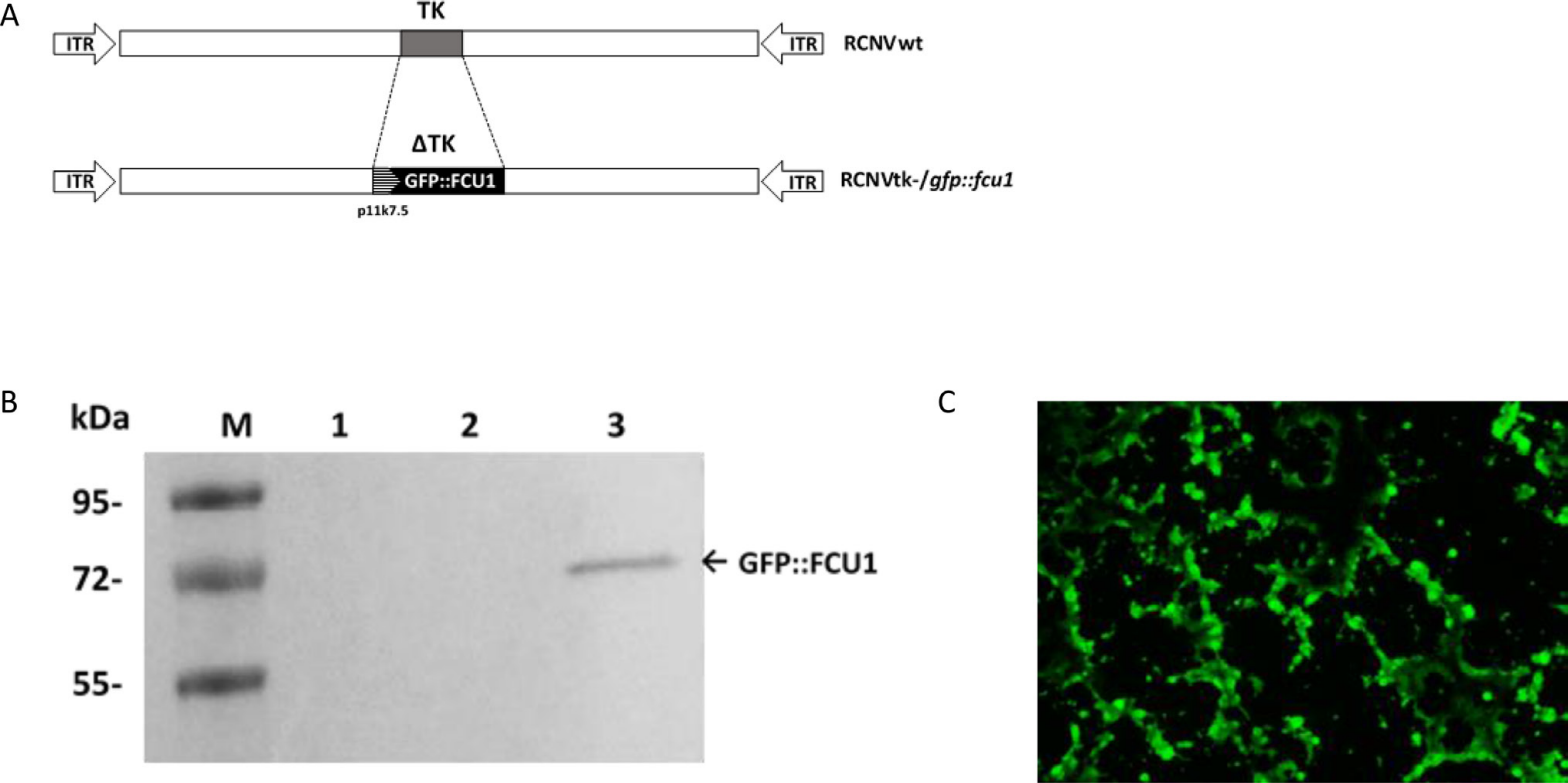
Generation of RCNV expressing the *GFP::FCU1* fusion gene and evaluation of the GFP::FCU1 protein expression (**A**) Schematic representation of Raccoonpox viruses used in this study. RCNtk-/*gfp::fcu1* contains a deletion of *TK* gene and insertion of a fusion gene between *eGFP* and *FCU1* genes. The *GFP::FCU1* fusion gene is driven by the synthetic p11k7.5 promoter. (**B**) Western blot detection of the GFP::FCU1 protein by anti-FCU1 monoclonal antibody. Lane 1 (left to the right), mock-infected LoVo cells; lane 2, LoVo cells infected with RCNVwt; lane 3, LoVo cells infected with RCNtk-/*gfp::fcu1*. Molecular weight standards are shown in kDa on the left. The presence of GFP::FCU1 fusion protein (*M*_r_ 72,000) is indicated (arrow). (**C**) Fluorescent microscopy showing the GFP::FCU1 protein expression. HCT 116 cells were infected with RCNtk-/*gfp::fcu1* at MOI 0.001 and transgene expression (GFP) was monitored 72 h post infection by fluorescent microscopy.

A TK-deleted recombinant Raccoonpox virus expressing the *GFP::FCU1* fusion gene, inserted in the *TK* locus, was generated from the wild type RCNV (RCNVwt). A shuttle plasmid which expressed the *GFP::FCU1* gene regulated by the VACV synthetic p11k7.5 promoter was used to insert the fusion gene into the *TK* locus by homologous recombination, creating the TK-deleted RCNV, RCNtk-/*gfp::fcu1*. Genomic structures of wild-type and recombinant RCNtk-/*gfp::fcu1* are shown in Figure 2A. A western blot using mouse monoclonal antibody directed against FCU1 confirmed the expression of *GFP::FCU1* protein (Figure 2B) with a band at 72 kDa as expected, demonstrating that RCNV polymerase can utilize a VACV promoter.

The ability of the recombinant RCNV to express a transgene and to spread within the culture was also monitored by GFP fluorescence at low MOI in the highly-RCNV-susceptible HCT 116 cell line. Abundant GFP-positive cells were present in the RCNtk-/*gfp::fcu1* infected culture despite the low MOI of 10^–3^ (Figure 2C), and due to viral replication, cytopathic effect was detectable compared with uninfected cells (not shown).

### Analysis of the FCU1 enzymatic assays and bystander effect

Expression of functional *FCU1* by RCNtk-/*gfp::fcu1* was next confirmed by quantification of the enzymatic activities of *FCU1*. The CDase and UPRTase activities were determined 48 h post infection by the analysis of the enzymatic conversions of 5-fluorocytosine (5-FC) to 5-fluorouracil (5-FU), and 5-FU to 5-fluorouridine-5’-monophosphate (5-FUMP), respectively, using lysates prepared from human LoVo tumor cells infected at an MOI of 10^–2^ by RCNV and RCNtk-/*gfp::fcu1.*

As shown in Table 1, CDase and UPRTase activities were found in cells infected with RCNtk-/*gfp::fcu1*, while no CDase and UPRTase activities were detectable in mock or RCNV-infected cells.

**Table 1:** Specific CDase and UPRTase activities in LoVo cell line

Vector	CDase 5-FC → 5-FU	UPRTase 5-FU → 5-FUMP
Mock	ND	ND
RCNVwt	ND	ND
RCNtk-/*gfp::fcu1*	17 ± 1.2	0.71 ± 0.2

CDase and UPRTase activities are expressed as the number of nanomoles of 5-FC deaminated per min per mg of protein and the number of nanomoles of 5-FU phosphorylated per min per mg of protein, respectively. The indicated enzymatic activities were measured as described in Materials and Methods. Each value represents the average of three independent experiments ± SD.

Abbreviations: CDase, cytosine deaminase; 5-FC, 5-fluorocytosine; 5-FU; 5-fluorouracil; 5-FUMP, 5-fluorouridine-5’-monophosphate; ND, not detectable; UPRTase, uracil phosphoribosyltransferase

A major strength of any prodrug activation model is the potential to extend the cytotoxic therapeutic effect to untransduced cells. In the case of FCU1/5-FC combination, an efficient bystander effect has been reported as 5-FU can reach neighboring cells by simple diffusion [36].

**Figure 3:**
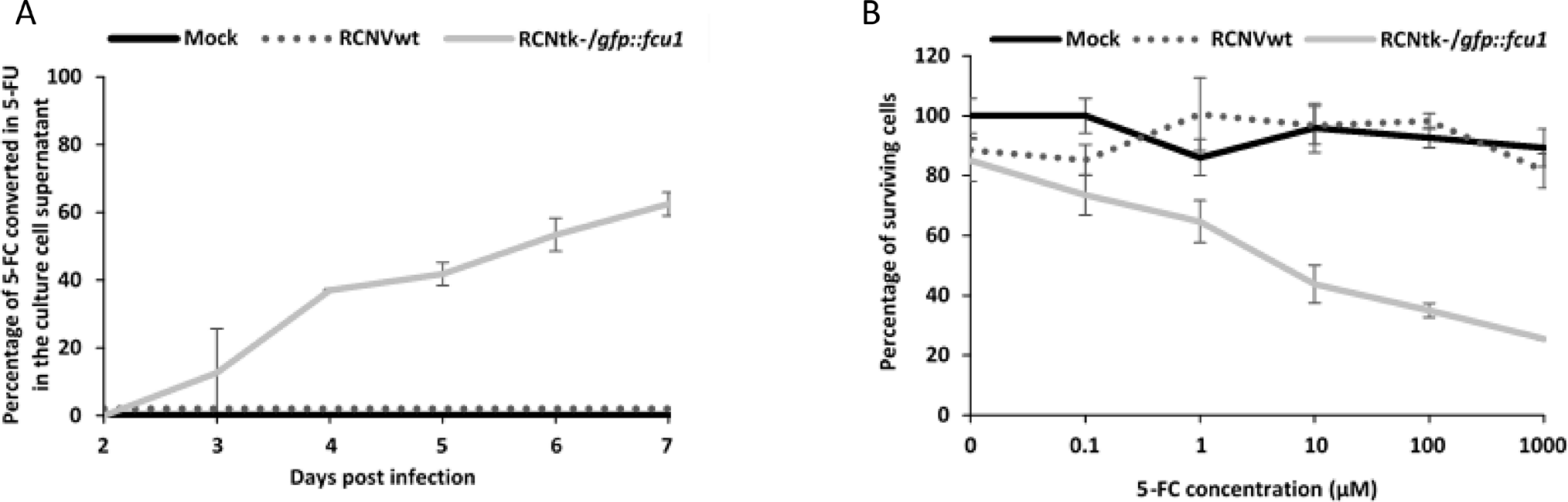
FCU1 functionality after expression by recombinant RCNV (**A**) Conversion of 5-FC to 5-FU and release of 5-FU in the cell culture supernatant. LoVo cells were infected with the indicated vector at a MOI of 10^–3^ and then incubated with 0.3 mM 5-FC from day 2 to day 7 post infection. The detection and the relative concentration of 5-FC and 5-FU in the culture supernatant was measured by HPLC. The results are expressed as the percentage of 5-FU released relative to the total amount of 5-FC+5-FU. Each data point represents the mean of triplicate determinations ± SD. (**B**) *In vitro* sensitivities of infected human tumor cells to 5-FC. LoVo human tumor cells were infected with the RCNVwt and RCNtk^-^/*gfp::fcu1* at a MOI of 10^–4^. After 48 h, cells were grown in the presence of increasing concentrations of 5-FC. Cell survival was determined 3 days later as described in Materials and Methods section. Cell viability results are expressed as the percentage of viable cells relative to untreated/non-infected cells. Each data point represents the mean of triplicate determinations ± SD.

The analysis of LoVo cell supernatants by high-pressure liquid chromatography (HPLC) showed a progressive release of 5-FU in extracellular medium of LoVo cells transduced with RCNtk-/*gfp::fcu1* at MOI of 10^–4^ and incubated with 0.3 mM 5-FC (Figure 3A). Five days after 5-FC treatment, approximately 65% of 5-FC was converted into 5-FU in supernatants of RCNtk-/*gfp::fcu1* LoVo-infected cells whereas no 5-FU was detected in supernatants of RCNV-infected cells confirming functionality and efficacy of the FCU1 protein expressed by the recombinant RCNtk-/*gfp::fcu1* vector. Together, these *in vitro* enzymatic activities demonstrate that RCNV can express a functional therapeutic *FCU1* protein and is an efficient vector for viral directed enzyme prodrug therapy (VDEPT).

### Cell killing by combination of prodrug activation with viral oncolysis

We next compared the antiproliferative activity of RCNtk^-^/*gfp::fcu1* vector alone or combined with 5-FC treatment. RCNVwt or RCNtk^-^/*gfp::fcu1* were used to infect LoVo cancer cells at an MOI of 10^–4^, corresponding to low cytotoxicity. After 48 h, 5-FC was added to the cultures at a range of concentrations, and cell viability was determined 3 days later by trypan blue exclusion. As shown in Figure 3B, oncolytic activity of both RCNVwt and RCNtk^–^/*gfp::fcu1*, in the absence of 5-FC prodrug, were similar and resulted in a low antiproliferative effect (10% of cytotoxicity). The addition of 5-FC had no impact on the viability of mock and RCNV-infected tumor cells. In contrast, the 5-FC conferred increased cytotoxicity in a prodrug dose-dependent manner to RCNtk-/*gfp::fcu1*-infected tumor cells. The combination of a low amount of RCNtk-/*gfp::fcu1* with 1 mM of 5-FC induced 75% mortality of LoVo cells. These results indicate that, following conversion of the prodrug 5-FC to the cytotoxic agent 5-FU, recombinant RCNtk-/*gfp::fcu1* acquired an enhanced *in vitro* anti-tumor activity in the presence of 5-FC.

### Recombinant RCNV infects and replicates selectively in human tumor cells *in vitro*

**Figure 4:**
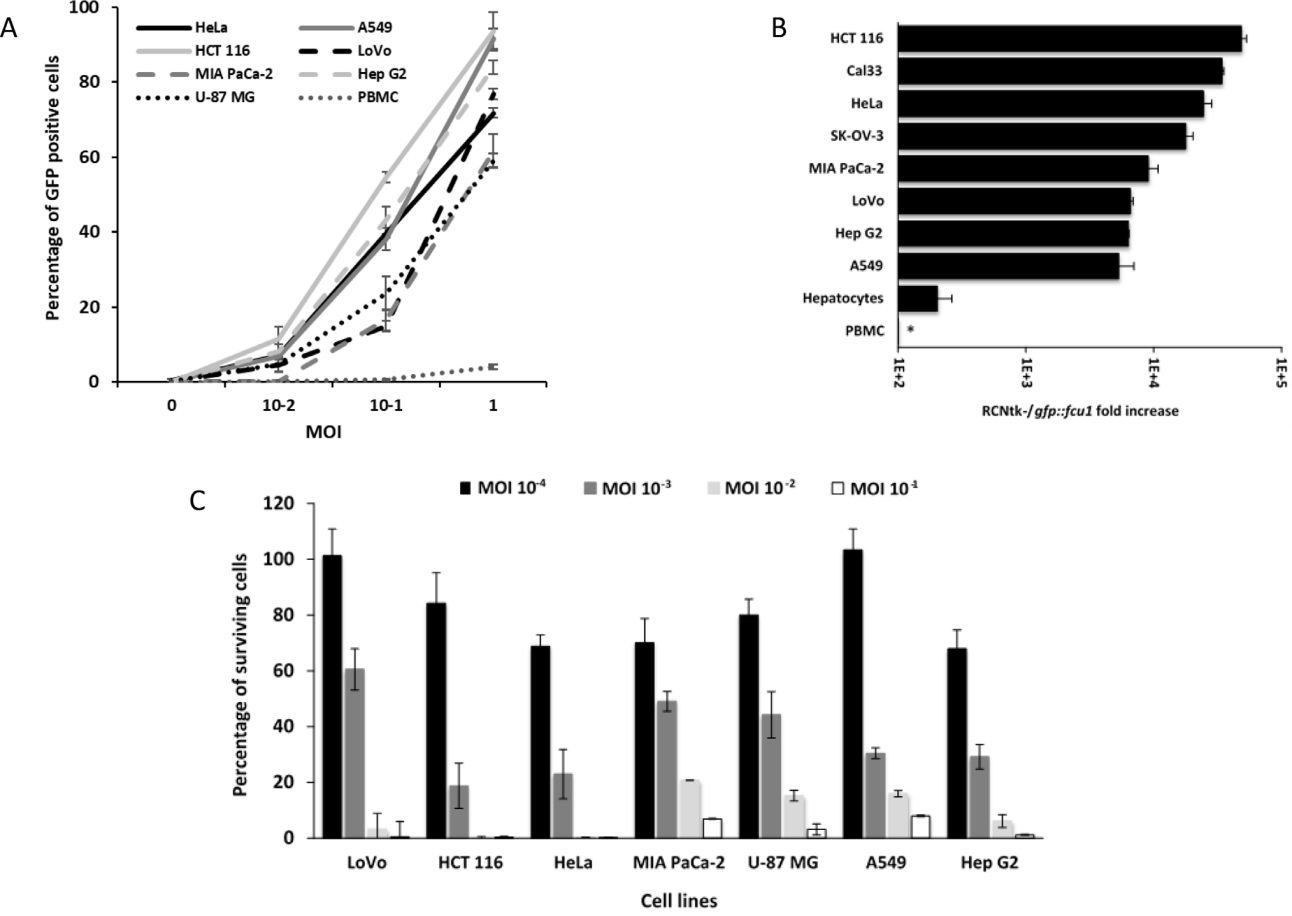
Infection, replication and oncolytic activity of RCNtk-/*gfp::fcu1* (**A**) Infection susceptibility of human cells to RCNtk-/*gfp::fcu1*. Cells were infected at the indicated MOI with RCNtk-/*gfp::fcu1* and the percentage of GFP-positive cells was determined by flow cytometry at 16 h post infection. The results were obtained from three separated experiments ± SD. (**B**) Replication in tumor cell lines and in primary human cell. Cells were infected at MOI 10^–3^, except PBMC at MOI 10^–1^, and harvested after 3 days of infection. Results are expressed as viral fold amplification and were obtained from three separated experiments ± SD. Asterisk denotes absence of amplification. (**C**) Oncolytic activities of RCNtk-/*gfp::fcu1* by measuring the cell viability 5 days after infection of different cancer cell lines. Tumor cells were infected at a MOI ranging from 10^–4^ to 10^–1^ and cell viability was determined by ViCell cell counter automate based on trypan blue exclusion method. Each data represents the mean of triplicate determinations ± SD.

The infectivity of the RCNtk^-^/*gfp::fcu1* virus in numerous cancer cell lines was determined, using a range of MOIs, by measuring the percentage of GFP positive cells at an early time point after infection, before cell death occurred. The results are summarized in Figure 4A. All cell lines tested were susceptible to infection by RCNtk^-^/*gfp::fcu1*. The transduction efficiency of this vector varied according to the human cancer cell lines tested, but all these cell lines showed more than 60% transduction efficiency at an MOI of 1. Cells highly susceptible to infection showed 40% to 60% transduction efficiency at MOI 0.1 (e.g. HCT 116, Hep G2, HeLa and A549). For cell lines that are less susceptible to infection, 10 to 20% of cells were GFP positive 16 h after RCNtk^-^/*gfp::fcu1* infection at MOI 10^–1^ (e.g. U-87 MG, Mia PaCa-2 and LoVo).

To evaluate the ability of RCNtk-/*gfp::fcu1* to replicate in tumor and in normal primary cells, we measured the level of viral particles produced after 72 h of infection in various tumor cell lines and in primary human hepatocytes. Viral titers calculated 72 h after infection at MOI 10^–3^ showed that most of the tumor cell lines supported RCNtk-/*gfp::fcu1* replication (Figure 4B). A549 and Hep G2 showed the lowest output of progeny virus, while higher titers were obtained in HCT 116 and CAL33. RCNtk-/*gfp::fcu1* replication resulted in at least 5,000-fold increase in all 8 tumor cell lines. In contrast, low amplification was observed in human primary hepatocytes, where RCNtk-/*gfp::fcu1* viral replication reached a maximum of 200-fold increase 72 h post infection (Figure 4B). In the same human primary hepatocytes, replication of RCNVwt resulted in at least 650-fold increase 72 h post infection (not shown) indicating the benefit of deleting *TK* gene for safety improvement. We also demonstrated that human peripheral blood mononuclear cells (PBMC) were weakly infected by the recombinant TK-deleted RCNV and that the virus was not able to replicate in these primary cells (Figure 4B). As shown in Figure 4A, after 16 h of infection, RCNtk-/*gfp::fcu1* poorly infected PBMC, with less than 5% of cells infected at MOI 1 and no viral amplification was observed 3 days post infection (Figure 4B). The replication of the recombinant RCNV was totally abortive in these blood cells.

These results indicated that the recombinant virus replicated efficiently in tumor cell lines but is significantly attenuated in normal primary cells, thus displaying a good therapeutic index.

### Tumor cell viability after RCNtk-/*gfp::fcu1* infection *in vitro*

A panel of 7 human cancer cell lines was used to evaluate the anti-tumor potency of RCNtk-/*gfp::fcu1* at various MOIs. Cell viability confirmed that RCNtk-/*gfp::fcu1* was able to kill all tested tumor cell lines of different origins with a dose-dependent effect (Figure 4C). Five days post infection and whatever the tested cell line, more than 95% of tumor cells were killed at a MOI of 10^–1^. RCNtk-/*gfp::fcu1* showed an efficient tumor cell killing activity on HCT 116 and HeLa cells with more than 75% lethality at a low MOI of 10^–3^.

### Antitumor effect in a human xenograft colorectal tumor model

**Figure 5:**
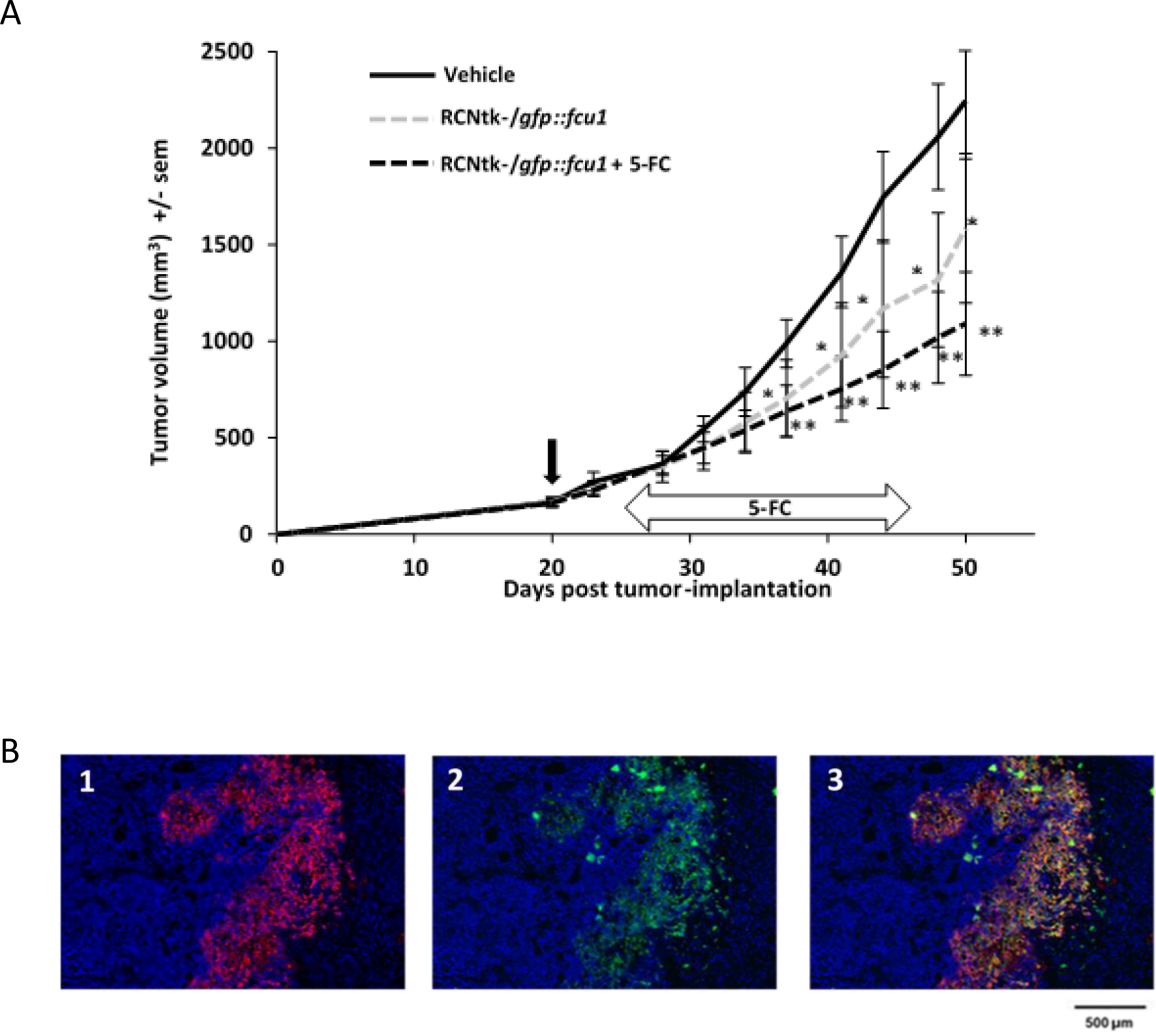
*In vivo* RCNtk-/*gfp::fcu1* anti-tumor efficacy in a colorectal xenograft model (**A**) Growth evolution of human colorectal LoVo tumors. Swiss nude mice were implanted subcutaneously with LoVo colorectal cancer cells. Mice (*n* = 10/group) were injected IT with RCNtk-/*gfp::fcu1* (10^7^ pfu) on day 20 after tumor cell transplantation. Five days after viral injection, 5-FC was administered by gavage at 200 mg/kg/day for three weeks. Mice were monitored until sacrifice based on high tumor volume. Vertical black arrow indicates the time of virus injection and horizontal arrow indicates the duration of 5-FC treatment. Single star and double stars represent, respectively, *P* < 0.05 and *P* < 0.01 compared to the control group (vehicle). Results are expressed in mean tumor volume ± sem. (**B**) Immunodetection of FCU1 and RCNV proteins in the LoVo xenograft tumors. Immunostaining of the tumor was performed, as described in Materials and Methods, five days after IT injection of RCNtk-*/gfp::*fcu1 at 1 × 10^6^ pfu. Cellular DNA was stained in blue with DAPI (1, 2 and 3), GFP::FCU1 protein was stained in red (1 and 3), and virus was stained in green (2 and 3). The merged picture is presented in 3.

*In vivo* efficacy of the virus was assessed in a human colorectal cancer model. Nude mice bearing established s.c. LoVo tumors (100–300 mm^3^) were treated with a single intratumoral (IT) injection of RCNtk-/*gfp::fcu1* at 1 × 10^7^ pfu. The 5-FC treatment started 5 days after virus injection with daily oral gavage at the dose of 200 mg/kg/day. A single IT injection of the recombinant RCNV in the LoVo colorectal model tumor resulted in a significant antitumoral effect, with a reduction of 35% of tumoral mass after 50 days as compared to the control group (*P* < 0.05; Figure 5A). The administration of 5-FC enhanced the antitumor activity of RCNtk-/*gfp::fcu1* resulting in a reduction of 55% in tumor growth as compared to the control group (*P* < 0.01). The addition of 5-FC led to an additional but not significant antitumor effect as compared to RCNtk-/*gfp::fcu1* alone. In previous studies, in the same s.c. Lovo model, it has been shown that 5-FC alone has no effect on tumor growth [4, 37]. Control experiments were also previously performed to determine the *in vivo* antitumor effect of 5-FU in non-transduced LoVo tumor cells. Despite the administration of doses of 5-FU that were at the maximum tolerated concentrations (i.p. injection of 10 mg of 5-FU per kg twice daily for 2 weeks), no statistically significant inhibition of tumor growth was observed [4, 37]. The improved antitumor effect of RCNtk-/*gfp::fcu1* combined with 5-FC was presumably due to the local production of high concentrations of 5-FC derivates via the *FCU1* gene product expressed from the p11K7.5 promoter.

### Detection of RCNV and FCU1 protein in the tumor

The presence of RCNV and the expression of *GFP::FCU1* gene in the tumor was analyzed by immunostaining of LoVo human colorectal tumor-bearing mice treated IT by the recombinant RCNV vector. FCU1 and viral immunostaining were performed five days after virus administration. Fluorescence microscopy of tumor sections confirmed the expression of FCU1 (Figure 5B1) and the presence of viral proteins (Figure 5B2). As expected, the distribution of the FCU1 fusion protein correlated with the area of virus detection (Figure 5B3). Virus was detected in numerous sites within the tumor mass showing that RCNV could replicate and rapidly invade the tumor.

## DISCUSSION

The primary objective of this study was to test the potential of non-vaccinia poxviruses as additional oncolytic virotherapies for the treatment of cancer. Raccoonpox virus, the most potent of the tested poxviruses, was modified to increase the oncolytic properties of this virus.

Few studies have been conducted that directly compare the oncolytic activity of different poxviruses. In *in vitro* studies comparing the oncolytic properties of different poxviruses, it has been shown that vaccinia was superior to Myxoma, Tanapox and Raccoonpox viruses for the control of head and neck squamous cell carcinoma and anaplastic thyroid cancer [38, 39].

In our study, a panel of different human cancer cells was used to compare the oncolytic potency of non-vaccinia poxviruses. Of the ten viruses used, five had modest or no basal oncolytic activity. FPV, a member of the *Avipoxvirus* genus, and SQFV, a member of the *Leporipoxvirus* genus, were unable to kill human tumor cells at an MOI ≤ 1. Avipox viruses are known to infect human cells but no progeny production occurs [40]. SQFV seems to have a very specific host range and exerts its viral pathogenic nature only in a certain squirrel species without affecting other non-squirrel species [41]. The oncolytic efficacy of SWPV, CTV, PCPV and BPSV was modest, with ED50 at a MOI of 10^–1^ for some cell lines. Regarding SWPV, CTV, and BPSV, natural infections in human have not been reported unlike PCPV that causes infection in humans [20]. From this study, four viruses emerged as potential oncolytic agents: YLDV, MYXV, ORFV and RCNV. The potential oncolytic properties of these 4 viruses were previously described. A wild type ORFV was able to replicate in a spectrum of human cancer cell lines and was therapeutically active in a human lung cancer xenograft model [9]. It has also been demonstrated that ORFV induced an antitumor response in syngeneic mouse models of cancer that is mediated largely by the potent activation of both cytokine-secreting, and tumoricidal natural killer cells [9]. A YLDV strain, derived from a lesion on a monkey caretaker, was genetically modified by inserting *GFP* into the *TK* site of the viral genome [11]. This recombinant YLDV was able to replicate in human tumor cell lines *in vitro* and was, after systemic injection in a human ovarian cancer xenograft model, able to preferentially replicate in tumor tissues [11].

MYXV has been extensively tested for its safety and oncolytic potential in both immunocompetent and immunocompromised murine models bearing various types of solid tumors, including glioblastoma, medulloblastoma, melanoma, and pancreatic cancer [25]. MYXV downregulates class I major histocompatibility complex (MHC) expression on the surface of infected cells [42]; a study demonstrated this effect in infected glioma cells *in vivo* [43]. This downregulation led to increased natural killer (NK) cell–mediated recognition and efficient killing of infected glioma cells [43]. Thus, MYXV infection not only leads to the direct killing of cancer cells but also promotes early immune cell–mediated antitumor responses.

We demonstrate here that RCNV is clearly the most potent oncolytic virus with consistent oncolytic potency among the tumor cell lines that we tested. A RCNV strain derived from a cat was previously genetically modified by inserting *GFP* into the *TK* site of the RCNV viral genome [10]. The recombinant RCNV replicated in the majority of human tumor cells tested and a significant efficacy of this virus was demonstrated in treatment of both xenograft and syngeneic models of solid tumors in mice [10].

Despite the strong efficiency of the recombinant virus *in vitro*, complete infection and lysis of the entire tumor is difficult. Therefore, oncolytic viruses are often armed. One example of arming includes enzyme-prodrug systems which can exert a strong bystander effect, and which may enhance the antitumor activity of the virus therapy by eliminating surrounding uninfected tumor cells. The FCU1/5-FC enzyme-prodrug system has been extensively investigated *in vitro* and in preclinical models of xenografts using a variety of delivery systems including replication-defective viruses [36, 37] and replication-selective oncolytic viruses [4, 6, 13, 44–48]. The proof of this suicide gene concept has also been demonstrated in human using TG4023, a non-propagative VACV (MVA) hosting the *FCU1* gene [49]. In this phase I study, after a single percutaneous IT injection of TG4023 in primary or metastatic liver tumors in combination with systemic administration of 5-FC, therapeutic 5-FU concentrations in tumors were detected without significant systemic exposure to the cytotoxic anticancer drug.

Here we have shown that the recombinant RCNV efficiently infects and expresses the *GFP::FCU1* marker/arming fusion gene in numerous cancer cell lines. The *FCU1* gene, through its expression by RCNtk-/*gfp::fcu1*, was able to convert non-toxic prodrug 5-FC into cytotoxic 5-FU and 5-FUMP. The produced 5-FU diffuses in and out of cells and does not require cell-to-cell contact for cell toxicity. This bystander effect enhances the antitumor efficacy of the virus directed enzyme pro-drug therapy approach by eliminating surrounding uninfected tumor cells *via in-situ* production of cytotoxic 5-FU [50]. In a human colorectal cancer *in vitro* model, the recombinant RCNtk-/*gfp::fcu1* vector exhibited less than 20% cytotoxicity but reached more than 75% cytotoxicity upon addition of 5-FC and production of 5-FU in the culture media. In addition, in comparison with the parental RCNVwt, we have noted that the TK-deficient virus displayed reduced multiplication in human primary hepatocytes indicating that this *TK* deletion, as for other member of the *Orthopoxvirus* genus, reduces viral pathogenicity [8, 13]. This TK-deleted virus replicated efficiently in human tumor cells and was notably attenuated in normal primary cells, which gives it a high therapeutic index (defined as the ratio of viral replication in tumor cells to that in primary cells).

Therapeutic activity of the recombinant RCNV was evaluated *in vivo* in a human xenograft colorectal tumor model. In this model, after a single intratumoral administration of RCNtk-/*gfp::fcu1*, we demonstrated that RCNtk-/*gfp::fcu1* plus 5-FC produced a superior antitumor effect than either agent acting alone. Our data showed a benefit in combining the oncolytic virotherapy using RCNtk-/*gfp::fcu1* and the prodrug 5-FC. However, 5-FC treatment can still be optimized. It might be possible, in order to increase the efficacy of the combination, to start earlier the 5-FC treatment (eg start of 5-FC administration 3 days post injection instead of 5 days post injection). It should be noted that only one cycle of RCNtk-/*gfp::fcu1* plus 5-FC was administered and we can postulate that a second cycle could increase the antitumor efficacy of the treatment. It would be important in this context to know whether the expression of *FCU1* and viral genes in injected tumors persists in residual growing lesions beyond the single early time-point tested (5 days post injection). A second administration of the virus would be possible, knowing that no clinical signs of illness (death, weight loss, lethargy and hyperactivity) were observed following inoculation of the recombinant RCNV. Moreover, no skin lesions, characteristic of poxvirus infection, were detected. This absence of adverse effects was corroborated by the weak replication of the recombinant RCNV in primary hepatocytes and its abortive replication in PBMC. Previous studies have shown that PBMC were also weakly infected by the VACV Pexa-Vec (JX-594) and that this VACV was not able to replicate in these primary cells [51].

In addition of the direct tumor cell lysis, a second mechanism by which oncolytic vectors mediate tumor cell destruction is *via* induction of nonspecific and specific antitumor immunity [8]. To examine RCNV interaction with the immune system, future works would be to evaluate the oncolytic potential of the recombinant RCNV in syngeneic immunocompetent models.

VACV is the most commonly used oncolytic poxvirus vector, however, its use is limited by its potential virulence, especially in immunocompromised hosts. ORFV and YLDV could infect human tissues and induce skin lesions even with low amount of viruses [52]. On the other hand, MYXV is extremely safe for human but some antitumor effects were only observed with high doses and repeat administration of virus [53]. RCNV is a member of the *Orthopoxvirus* genus, with no known pathogenicity in any mammalian species so far [54, 55]. The safety of RCNV in human remains unknown, but a case of human infection with a recombinant RCNV expressing rabies virus glycoprotein suggests that it is harmless for human [56]. Moreover, in contrast to other double stranded DNA viruses, poxviruses, including RCNV, encode their own DNA replication and transcription machinery and they replicate in the cytoplasm, avoiding the risk of integration into the host genome.

The RCNV strain used in this study was originally isolated from apparently healthy raccoons [30] and has been shown to be avirulent in numerous animal models. Recombinant RCNV vaccines have been successfully employed in mice, raccoons, skunks, foxes, bobcats, rabbits, domestic cats, piglets, sheep, bats and non-human primates [55, 57–62]. RCNV-vectored rabies vaccine has also been administered *via* oral, intranasal and conjunctival routes as a mucosal vaccine in cats and was found to be safe [60]. A rabies–RCNV vaccine has been approved by the US Department of Agriculture. It was recently reported that RCNV Herman strain is less virulent and much safer than VACV in immunocompromised or pregnant mouse models [54]. Although the wild type Herman strain was highly attenuated, deletion of *TK* gene attenuated it further [29], as is the case in VACV [63].

RCNV was attenuated compared to VACV, the best-known member of the *Orthopoxvirus* genus, although it still replicated well in human tumor cells. Ten genes conserved in most orthopoxviruses are missing in RCNV and might individually or in combination explain the profound lack of virulence of RCNV. The majority of these genes encode proteins that are implicated in virulence [29]. Like the other members of the *Orthopoxviridae* family, RNCV possesses essential characteristics of use in virotherapy: an easily modifiable genome that offers the possibility to transform its phenotype and to insert large therapeutic foreign genes, cytoplasmic replication with no risk of genomic integration, ability to replicate in human tumor cells, rapid lytic cycle and no pre-immunity that might hinder vector replication. Furthermore, RCNV is genetically and likely immunologically more distant from VACV than other members of the *Orthopoxvirus* genus [29] so cross resistance could be weaker than homologous resistance. In this context, subjects immunized against VACV should respond better to treatment with an oncolytic virus based on RCNV than one based on VACV.

In summary, this study represents, to our knowledge, the first characterization of an oncolytic RCNV “armed” with a therapeutic transgene. Considering these results and the apparent nature of its non-pathogenicity in human species, RCNV could become a promising vector candidate for oncolytic viral therapies.

## MATERIALS AND METHODS

### Cell lines

Human colon cancer cell lines LoVo (CCL-229™) and HCT 116 (CCL-247™), human lung cancer cell line A549 (CCL-185™), hepatocarcinoma human cell line Hep G2 (HB 8065™), glioblastoma human cancer cell line U-87 MG (HTB-14™), cervix human cancer cell line HeLa (CCL-2™), pancreatic human cancer cell line MIA-Paca-2 (CRL-1420™), human ovarian cancer cell line SK-OV-3 (HTB-77™), embryonic swine kidney ESK-4 cell line (CL-184™), monkey kidney cell line Vero (CCL-81™), monkey kidney cell line CV-1 (CCL-70™), monkey kidney cell line BS-C-1 (CCL-26™), rabbit kidney RK13 cell line (CCL-37™), bovine turbinate BT cells (CRL-1390™) were obtained from the American Type Culture Collection (ATCC, Rockville, MD, USA). Human head and neck cancer cell line CAL33 was kindly provided by Dr. G. Milano (Centre Antoine-Lacassagne, Nice, France). All cell lines were grown in recommended media supplemented with 10% fetal calf serum (FCS). Primary chicken embryo fibroblasts (CEF) were prepared from chicken embryos obtained from fertilized eggs (Charles River SPAFAS) previously incubated 11 or 12 days at 37 °C in a humid atmosphere. Chicken embryos were dissected and treated with a 2.5% (*w*/*v*) solution of trypsin. CEF were maintained in Eagle-based Medium (MBE) supplemented with 5% fetal calf serum. Human peripheral blood mononuclear cells (PBMC) were obtained after buffy coat extraction from blood bag (EFS, Strasbourg, France). Fresh human hepatocytes were purchased from Biopredic International (Rennes, France) and maintained in the recommended hepatocyte medium provided by the supplier (Biopredic International).

### Viruses

All viruses except Fowlpox virus strain (FPV) were obtained from American Type Culture Collection (ATCC, Rockville, MD, USA): Raccoonpox virus strain Herman (VR-838™) (RCNV), Bovine Papular Stomatitis virus strain Illinois 721 (VR-801™) (BPSV), ORF virus strain NZ2 (VR-1548™) (ORFV), Pseudo-cowpox virus strain TJS (VR-634™) (PCPV), Myxoma virus strain Lausanne (VR-115™) (MYXV), Yaba-like disease virus (VR-937™) (YLDV), Swinepox virus strain Kasza (VR-363™) (SWPV), Cotia virus strain SP AN 32 (VR-464™) (CTV), Squirrel Fibroma virus strain Kilham (VR-236™) (SQFV). The Fowlpox virus strain FP9 was kindly provided by Pr Skinner. None of the viruses used is characterized by a biohazard rating greater than biosafety level 2. All viruses were produced on HeLa cells except for SWPV produced on ESK-4 cells and FPV produced on CEF.

### Virus titration

RCNV, MYXV, FPV, SQFV and YLDV were titrated by plaque assay on Vero cells, RK13 cells, CEF, CV-1 cells and BS-C-1 cells respectively. Titration by TCID50 was performed for BPSV and PCPV on BT cells, CTV and ORFV on BSC-1 cells and SWPV on ESK-4 cells.

### Engineering of a recombinant Raccoonpox virus expressing the *GFP::FCU1* fusion gene

A recombinant RCNV was created by insertion of the *GFP::FCU1* fusion gene into the RCNV thymidine kinase (TK) locus (RCNV CDS 090). Briefly, Vero cells were infected with RCNV at a MOI of 10^–2^ and incubated at 37° C for 2 h, then the infected-cells were transfected with a shuttle plasmid containing the *GFP::FCU1* fusion gene under the control of the synthetic p11k7.5 promoter and surrounded by the flanking sequence of the *TK* gene.

The cells were then incubated for 48 h at 37°C. Double recombination occurred between TK homologous regions in the shuttle plasmid and the wild type virus, resulting in the insertion of the *GFP::FCU1* fusion gene into the *TK* locus of the RCNV (RCNtk-/*gfp::fcu1)*. Recombinant virus was isolated from GFP-fluorescent plaques and submitted to additional plaque purification cycles on Vero cells. Virus sequence was confirmed by multiple PCRs and DNA sequencing. Final recombinant RCNtk-/*gfp::fcu1* vector was grown on HeLa cells, purified by centrifugation through a sucrose gradient and titrated on Vero cells by plaque assay.

### Poxvirus infections *in vitro*

All infections were performed in suspension by a 30-min incubation of cells with virus dilutions in 100 µL phosphate-buffered saline (PBS). Cells were then plated in complete fresh medium and analysis was performed at various times post infection. To determine the *in vitro* transduction efficiency, cells were infected with RCNtk^-^/*gfp::fcu1* at various MOIs and 16 h later, cell suspensions were analyzed by flow cytometry using a FACScan instrument (Becton Dickinson).

### Western blotting

LoVo tumor cells were infected by RCNV*wt* and RCNtk-/*gfp::fcu1* at an MOI of 10^–1^ and incubated for 24 h. Cell lysate proteins (30 µg as determined by using a Bio-Rad protein assay) were run on a 10% SDS-polyacrylamide gel electrophoresis (PAGE) gel under reducing conditions and transferred onto a nitrocellulose membrane. The membrane was incubated with anti-FCU1 mouse monoclonal antibody 3H1 [4], washed and incubated with anti-mouse secondary antibody coupled to horseradish peroxidase (Amersham, Les Ulis, France). Detection was done using enhanced chemiluminescence (Amersham).

### Enzymatic assays

CDase and UPRTase activities in LoVo cells were determined using 5-FC (Toronto Research Chemicals Inc., North York, Canada) and 5-FU (Sigma, Missouri USA) as substrates, respectively. LoVo human tumor cells (3 × 10^6^ cells) were infected by RCNV and RCNtk-/*gfp::*fcu1 vector at a MOI of 10^–2^. Fourty-eight hours later, CDase and UPRTase activities detection was performed by enzymatic assays as previously described [37]. 5-FC, 5-FU and 5-FUMP were isocratically separated using HPLC (supelcosil LC-18-S column and UV detection at 260 nm and 280 nm). For detection of CDase activity, the mobile phase was 50 mM phosphoric acid adjust to pH 2.1 with ammonium hydroxide. For detection of UPRTase activity, the mobile phase was 20 mM KH_2_PO_4_, 5 mM tetrabutylammoniumsulfate, 5% methanol adjusted to pH 5 with potassium hydroxyde.

CDase activity was also quantified by measuring the amount of 5-FU released in the culture media. LoVo cells were infected with the different vectors at a MOI of 10^–4^ and plated in 6-well culture dish (1 × 10^6^ cells/well). After 48 h, 0.3 mM 5-FC was added to the culture medium. Every day for 1 week, 5-FC and 5-FU concentrations in the media were measured by HPLC. Fifty µL of media were quenched with 50 µL of acetonitrile. The samples were vortexed and centrifuged. The organic supernatant was evaporated to dryness and reconstituted in 50 µl of water and analyzed by HPLC using a mobile phase of 50 mM phosphoric acid adjusted to pH 2.1. Results are expressed as the percentage of 5-FU relative to the total amount of 5-FC + 5-FU after various incubation times with 5-FC.

### *In vitro* cell sensitivity to 5-FC

LoVo tumor cells in suspension were infected by the indicated Raccoonpox viruses at a MOI of 10^–4^. A total of 3 × 10^5^ cells/well were plated in 6-well culture dishes in 2 ml of medium supplemented with 10% FCS. After 48 h of infection, cells were exposed to various concentrations of 5-FC ranging from 0.1 to 1000 µM. Three days later, cell viability was determined by trypan blue exclusion using a Vi-Cell cell counter (Beckton Dickinson, California). Results are expressed as percentage of viable cells, 100% corresponding to uninfected cells without 5-FC.

### Determination of virus-induced cell death

Tumor cells were infected by respective poxviruses at MOI of 1, 10^–1^, 10^–2^, 10^–3^, 10^–4^ and 10^–5^. A total of 3 × 10^5^ cells/well were plated in 6-well culture dishes in 2 ml of medium supplemented with 10% FCS. Cells were then cultured at 37 °C for 5 days and the viable cells were counted by trypan blue exclusion using a Vi-Cell cell counter (Beckmann Coulter, California). Results were expressed as percentage of viable cells, 100% corresponding to uninfected cells. For the evaluation of the oncolytic activity of the 10 poxviruses in 5 tumor cell lines, ED50 values were calculated by using the software GraphPad Prism (GraphPad Software, Inc.). ED50 was defined as the initial virus dose (MOI expressed in pfu/cell) that resulted in 50% cell viability at 5 days post infection as compared to untreated controls.

### *In vitro* virus replication

Growing human tumor cells were seeded onto 6-well plates at 5 × 10^5^ cells/well. Twenty four hours later, cells were infected with RCNtk-/*gfp::fcu1* at an MOI of 10^–3^ and incubated in fresh growth medium supplemented with 10% FCS. Before harvesting, pictures were taken under fluorescent microscopy for GFP expression visualization. Supernatants and cells were collected 72 h post infection and submitted to a quick freeze-thaw cycle and sonication to release intracellular viral particles. Viral titer in cell lysates was quantified on Vero cells by plaque assay. For the assessment of the replication activity of RCNV, ORFV, CTV and SQFV on U-87 MG, the cells were infected by the respective poxvirus at MOI 10^–2^ and viral titers were determined 24, 48 and 72 h post infection as described above.

### Animals

All animal protocols were carried out according to standard operating procedures of Felasa and have been approved by the French Research and Education Ministry (APAFIS#7049-2016060816539934 v6).

### *In vivo* antitumor activity of the recombinant RCNV in subcutaneous tumor model

Female Swiss nude mice were obtained from Charles River Laboratories. Animals used in the study were uniform in age (6 weeks) and body weight (20–23 g).

Mice were injected subcutaneously (s.c.) into the right flank with 5 × 10^6^ human LoVo tumor cells. When tumors reached a diameter of 100–300 mm^3^, mice were assigned in a random, blinded manner to receive the recombinant RCNV. Nude mice were treated by a single intratumoral injection of RCNtk-/*gfp::fcu1* vector at the dose of 1 × 10^7^ pfu (in 100 µl PBS) or vehicle (control group). Five days post injection, 5-FC was administrated by oral gavage at 100 mg/kg (0.5 ml 5-FC 0.5% in water) twice a day for 3 weeks. Tumor size was measured twice a week using calipers. Tumor volume was calculated in mm^3^ using the formula (Π/6) (length × width^2^).

### Immunohistochemistry

Detection of FCU1 and viral proteins was performed by immunohistochemical labelling. For each treatment 3 slides were analyzed. Five days after intratumoral injection of RCNtk-/*gfp::fcu1* at the dose of 1 × 10^6^ pfu, resected LoVo tumors were fixed with 4% formaldhehyde in 0.1 M phosphate buffer. Tumors where then desiccated and embedded in paraffin wax. Sections (5 µM) were mounted on adhesive glass slides and used for histological analysis.

Detection of FCU1 protein was performed by immunostaining of the slides-fixed tumors with anti-FCU1 mouse monoclonal antibody 3H1 [4] followed by Goat anti Mouse-IgG-Polymer Dextran HRP (DAKO, K4001) (red staining). RCNV-infected cells were detected upon incubation of the slide with rabbit IgG anti vaccinia virus (B 65101R, Biodesign, dilution 1/1000) followed by Goat anti-Rabbit IgG-Polymer Dextran HRP (DAKO, K4003, dilution 1/1000) (green staining). To block non-specific antibody binding, slides were incubated 30 minutes with Linblock solution between the two staining steps. Coverslips were counterstained with DAPI (B-2883 SIGMA) (blue staining) and mounted on glass slides. Negative control tumors also underwent the same immunostaining treatment for comparison purposes. Slides were analyzed using Nikon microscopy.

### Statistical analysis

Statistical analyses of tumor volumes were performed using the nonparametric Mann–Whitney *U* test (Statistica 7.1 software, StatSoft, Inc.). A *P*-value < 0.05 was considered to be statistically significant.

## Abbreviations

5-FC: 5-fluorocytosine
5-FU: 5-fluorouracil
5-FUMP: 5-fluorouridine-5′-monophosphate
BPSV: bovine papular stomatitis virus
CDase: cytosine deaminase
CTV: Cotia virus
FPV: Fowlpox virus
HCC: hepatocarcinoma
MOI: multiplicity of infection
MYXV: Myxoma virus
ORFV: ORF virus
PCPV: Pseudo-cowpox virus
PFU: plaque forming units
PPVs: Parapoxviruses
RCNV: Raccoonpox virus
RCNtk-/gfp::fcu1: thymidine kinase-deleted Raccoonpox virus expressing the GFP::FCU1 fusion gene
RCNVwt: wild type Raccoonpox virus
SQFV: Squirrel fibroma virus
SWPV: Swinepox virus
TK: thymidine kinase
UPRTase: uracil phosphoribosyltransferase
VACV: Vaccinia virus
YLDV: Yaba-like disease virus
